# Prognostic Prediction, Immune Microenvironment, and Drug Resistance Value of Collagen Type I Alpha 1 Chain: From Gastrointestinal Cancers to Pan-Cancer Analysis

**DOI:** 10.3389/fmolb.2021.692120

**Published:** 2021-07-30

**Authors:** Yi Liu, Jinmin Xue, Maoxi Zhong, Zhi Wang, Jie Li, Yuxi Zhu

**Affiliations:** ^1^ Department of Oncology, The First Affiliated Hospital of Chongqing Medical University, Chongqing, China; ^2^ Department of Oncology, Jinshan Hospital of the First Affiliated Hospital of Chongqing Medical University, Chongqing, China; ^3^ Chongqing Clinical Cancer Research Center, The First Affiliated Hospital of Chongqing Medical University, Chongqing, China

**Keywords:** *COL1A1*, pan-cancer, prognosis, tumor immune microenvironment, gastrointestinal cancers

## Abstract

**Background:** Gastrointestinal cancers patients might experience multiple primary tumors in the digestive tract. Therefore, identifying potential biomarkers can help us better understand the underlying mechanism. From the GEO database, four profiles of gastrointestinal cancers were gathered for the screening process, and six hub genes were found by bioinformatics analysis. *Collagen type I alpha 1 chain* (*COL1A1*), one of the hub genes, is a component of the extracellular matrix and is critical for tumor microenvironment. However, the expression level, signaling pathway, prognostic prediction, and immunological value of *COL1A1* in different cancers remain unclear.

**Methods:** We comprehensively analyzed gene expression and genetic alteration patterns of *COL1A1* among 33 types of malignancies from The Cancer Genome Atlas (TCGA) and the Genotype-Tissue Expression projects. Besides, we explored the correlation of *COL1A1* with cancer prognosis, immune infiltrates, PD-L1, tumor mutational burden (TMB)/microsatellite instability status (MSI), and the pathway and drug sensitivity of co-expressed genes.

**Results:** The results showed that *COL1A1* was highly expressed and associated with poor prognosis in the majority of cancers. The most common alteration type of *COL1A1* was missense mutation, and *COL1A1* was associated with poor prognosis in KIRP, LGG, MESO, SKCM, and STAD. For the immunologic significance, *COL1A1* expression was closely related to high TMB in THYM, LAML, ACC, KICH, PRAD, and LGG, and high MSI in TGCT, MESO, PRAD, COAD, SARC, and CESC. In addition, *COL1A1* was positively correlated with the abundance of CAFs, macrophages, and tumor-infiltrating lymphocytes. However, it was negatively correlated with CD8^+^ T cells mainly in CESC, HNSC-HPV+, and SKCM. Besides, as a component of the extracellular matrix, *COL1A1* was involved in the activation of epithelial-mesenchymal transition (EMT), and high expression of *HTRA1* was resistant to multiple drugs.

**Conclusion:***COL1A1* can serve as a prognostic and immunological biomarker in different cancers.

## Introduction

Esophageal, gastric, and colorectal cancer are the most common types of gastrointestinal cancers. According to Global Cancer Statistics 2018, colon, gastric, rectal, and esophageal cancer have been ranked the fourth, sixth, eighth, and ninth, respectively, in the incidence of human malignant tumors, and their mortality rates were 5.8, 8.2, 3.2, and 5.3%, respectively (Bray et al., 2018). However, it has been found that gastrointestinal cancers patients might experience multiple primary tumors in the digestive tract (Yoshida et al., 2020; van de Ven et al., 2020). Furthermore, it was reported that several cases were related to synchronous or metachronous primary gastrointestinal tract malignancies (Bratislav et al., 2015; Yoshikawa et al., 2016; Kim et al., 2017; Arakawa et al., 2018). Hence, we identified some hub genes including Collagen *Type I Alpha 1 Chain* (*COL1A1*) of multiple primary tumors in the gastrointestinal tract based on bioinformatics technology, which might become potential diagnostic biomarkers of gastrointestinal cancers.

*COL1A1* is the gene which encodes the pro-alpha 1 chains of type I collagen whose triple helix comprises two alpha 1 chains and one alpha 2 chain (Prockop, 1990); the protein encoded by this gene is an important component of the extracellular matrix (ECM). *COL1A1* is known to be overexpressed in several cancers other than gastrointestinal cancers (Shi et al., 2015; Zhang et al., 2018; Yin et al., 2019), such as thyroid cancer (Huang et al., 2019), lung cancer (Grigoroiu et al., 2015), breast cancer (Liu et al., 2018b), and renal cancer (Boguslawska et al., 2016). However, there is no pan-cancer analysis to comprehensively elucidate the potential role of *COL1A1* in various tumor types. Thus we expand our research from gastrointestinal cancers to the pan-cancer analysis of *COL1A1*.

## Materials and Methods

### Microarray Data of Gastrointestinal Cancers

We downloaded four gene expression profiles from the GEO database (http://www.ncbi.nlm.nih.gov/geo) (Barrett et al., 2013): the GSE20347 of esophageal squamous cell carcinoma (ESCC), the GSE92396 of esophageal adenocarcinoma (EAC), the GSE103236 of stomach adenocarcinoma (STAD), and the GSE110224 of colorectal adenocarcinoma (CRAC). GSE203475 (Hu et al., 2010) was based on GPL571 ([HG-U133A_2] Affymetrix Human Genome U133A 2.0 Array) and contained 17 pairs of esophageal squamous cell carcinoma tissues and matched normal adjacent tissues. GSE92396 was based on GPL6244 ([HuGene-1_0-st] Affymetrix Human Gene 1.0 ST Array [transcript (gene) version]) and contained 12 esophageal adenocarcinoma tissues and 10 normal esophageal tissues. GSE1032366 (Chivu Economescu et al., 2010) was based on GPL4133 [Agilent-014850 Whole Human Genome Microarray 4 × 44K G4112F (Feature Number version)], which contained 10 pairs of cancerous and normal adjacent tissue from gastric adenocarcinoma patients. GSE110224 (Vlachavas et al., 2019) was based on GPL570 ([HG-U133_Plus_2] Affymetrix Human Genome U133 Plus 2.0 Array). It included 17 pairs of histologically confirmed colorectal adenocarcinoma tissues and normal adjacent tissues.

### Identification of the DEGs in Profile

GEO2R (http://www.ncbi.nlm.nih.gov/geo/geo2r) is a tool for analyzing differentially expressed genes in the GEO database, which can compare the expression of genes in tumor and normal samples. Adj. *p* < 0.05 and |logFC| > 1 were set as the cutoff criteria to select DEGs for these microarray, respectively. Then the overlapping DEGs among the four datasets were identified by the online Venn diagram tool (http://bioinformatics.psb.ugent.be/webtools/Venn/).

### Gene Ontology and Pathway Enrichment Analysis of DEGs

Database for Annotation, Visualization, and Integrated Discovery (DAVIDv6.8 https://david.ncifcrf.gov/) provides a comprehensive set of functional annotation tools to understand the biological meaning behind a large number of genes (Huang da et al., 2009). DAVID was employed to carry out gene ontology (GO) and the Kyoto Encyclopedia of Genes and Genomes (KEGG) analysis of DEGs. The GO and KEGG pathways were plotted by http://www.bioinformatics.com.cn, an online platform for data analysis and visualization.

### Hub Genes Screening From the PPI Network

The protein–protein interaction (PPI) network of differentially expressed genes was constructed based on the online website STRING (STRING; http://string-db.org) (version 11.0) (Szklarczyk et al., 2019) and was further illustrated by the Cytoscape software (Shannon et al., 2003). The MCODE plug-in in Cytoscape was utilized to identify key modules and hub genes. The preferred cutoff values were determined as degree cutoff values = 2, max. depth = 100, the node score = 0.2, and the k-score = 2.

### Gene Expression Analysis in Pan-Cancer

The Oncomine (https://www.oncomine.org) database is currently the world's largest cancer gene-chip database and an integrated data-mining platform, which can analyze differential gene expressions in normal and tumor tissues (Rhodes et al., 2004). Firstly, we used Oncomine to analyze the differential expression of *COL1A1* between tumor tissues and normal tissues. Next, the “Gene_DE” module of TIMER2 (tumor immune estimation resource, version 2) (http://timer.cistrome.org/) was employed to analyze the differential expression of *COL1A1* in different tumors and normal tissues (Li et al., 2020). For those tumors that lack normal or have a highly limited number of normal tissues, the “Expression analysis-Box Plots” module of GEPIA2 (Gene Expression Profiling Interactive Analysis, version 2) (http://gepia2.cancer-pku.cn/#analysis) (Tang et al., 2019) was used to obtain the expression difference between these tumor tissues and the corresponding normal tissues. In addition, we explored the *COL1A1* expression difference in different stages by the “Pathological Stage Plot” module of GEPIA2.

### Genetic Alteration Analysis

CBioportal (https://www.cbioportal.org) is an online database which provides visualization, analysis, and the ability to download large-scale cancer genomics data (Cerami et al., 2012). Herein, cBioportal was employed to obtain the alteration frequency and mutation type of *COL1A1* across all TCGA tumors. Then we explored the overall, disease-specific, progression-free, and disease-free survival differences with or without *COL1A1* genetic alteration of the tumor with the highest alteration frequency.

### Methylation Profile of *COL1A1*

UALCAN is a comprehensive and interactive web resource for analyzing cancer OMICS data (Chandrashekar et al., 2017). We investigated the *COL1A1* promoter DNA methylation level in gastrointestinal cancers and some certain types of cancer by UALCAN. MEXPRESS is a web tool which can visualize DNA methylation, expression, and clinical data (Koch et al., 2015). MEXPRESS was employed to determine the association between *COL1A1* DNA methylation and clinical data.

### Survival Prognosis Analysis

We used the “Survival Map” module of GEPIA2 to analyze the overall (OS) and disease-free survival (DFS) of *COL1A1* among all tumors. Furthermore, the “Survival Analysis” module was used to further analyze the survival outcome of the specific type of tumor.

### Immune Infiltration Analysis

The occurrence and development of tumors are closely related to the tumor immune microenvironment. We used the “Immune-Gene” module of TIMER2 to analyze the relationship between *COL1A1* expression and cancer-associated fibroblasts, CD8^+^ T cells, and macrophages. TISIDB (http://cis.hku.hk/TISIDB/index.php) is an integrated repository portal for tumor and immune system interaction, which integrates multiple heterogeneous data types (Ru et al., 2019). We used TISIDB to analyze *COL1A1* expression and tumor-infiltrating lymphocytes (TILs), immunoinhibitors, immunostimulators, and the MHC molecule.

### PD-L1, TMB, and MSI in Pan-Cancer

PD-L1, TMB, and MSI are important predictive markers of immunotherapy. We used GEPIA to analysis the association of *COL1A1* expression and PD-L1 (CD274) among different cancers. RNA-seq data of 33 types of tumor were downloaded from the Cancer Genome Atlas (TCGA) Genomic Data Commons (GDC) data portal website (https://portal.gdc.cancer.gov/). TMB is derived from the article “The Immune Landscape of Cancer” published by Thorsson et al. (2019); MSI is derived from the “Landscape of Microsatellite Instability Across 39 Cancer Types” article published by Bonneville et al. (2017). R software v4.0.3 was utilized for statistical analysis. If not otherwise stated, the rank-sum test detected two sets of data, and *p*-value <0.05 was considered statistically significant.

### Genes Co-expressed With *COL1A1* in Pan-Cancer: Pathway and Drug Sensitivity Analysis

We used the STRING database (https://string-db.org/) (Szklarczyk et al., 2019) to obtain the top 50 proteins most relevant to *COL1A1* protein expression. The parameters were set as follows: a minimum required interaction score [“Low confidence (0.150)”], meaning of network edges (“evidence”), max number of interactors to show (“no more than 50 interactors” in first shell), and active interaction sources (“Experiments”). Moreover, we used the GEPIA2 database to obtain the top 100 genes most correlated to *COL1A1*. Then we drew the Venn diagram to obtain the overlapping genes by the online Venn diagram tool (http://bioinformatics.psb.ugent.be/webtools/Venn/). Next, we used GSCALite (http://bioinfo.life.hust.edu.cn/web/GSCALite/) (Liu et al., 2018a) to conduct a pathway and drug sensitivity analysis on *COL1A1* and the obtained genes.

## Results

### Identification of DEGs in Gastrointestinal Cancers

**FIGURE 1:**
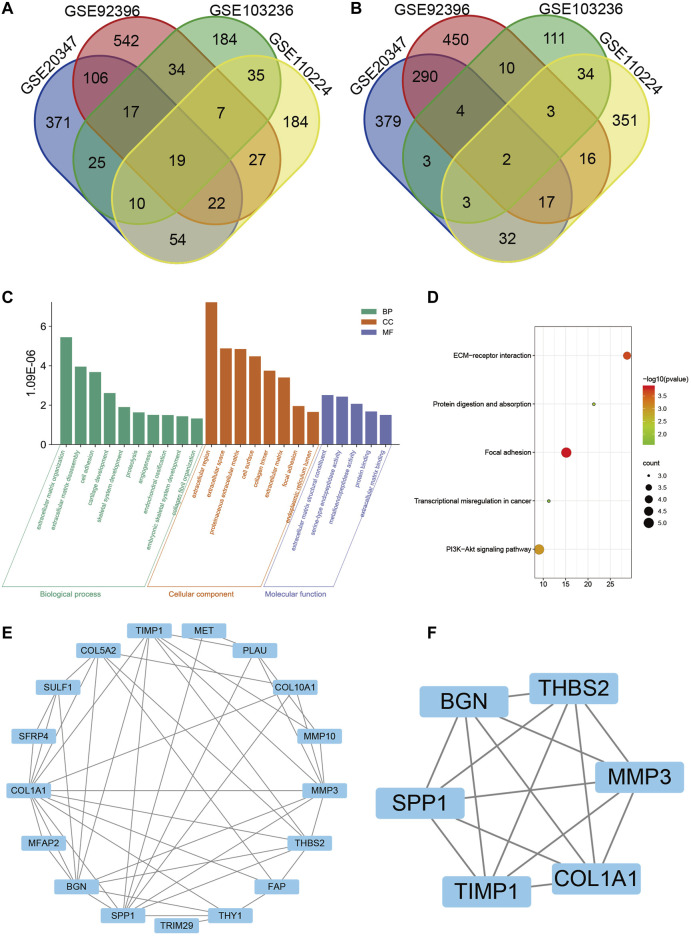
Identification of 21 differentially expressed genes (DEGs) from four microarrays (GSE20347, GSE92396, GSE103236, and GSE110224) of gastrointestinal cancers, among which 19 were upregulated and 2 were downregulated. **(A)** Upregulated genes. **(B)** Downregulated genes. **(C)** Gene Ontology (GO) analysis of DEGs, including biological process (BP), cellular component (CC), and molecular function (MF), respectively. **(D)** Kyoto Encyclopedia of Genes and Genomes (KEGG) pathway analysis of DEGs. **(E)** Protein–protein interaction (PPI) network of DEGs by Cytoscape. **(F)** Interaction network of hub genes, including *SPP1*, *BGN*, *THBS2*, *MMP3*, *COL1A1*, and *TIMP1*.

Based on the cutoff criteria, four mRNA expression profiles of esophageal squamous cell carcinoma, esophageal adenocarcinoma, gastric adenocarcinoma, and colorectal adenocarcinoma were gathered from the GEO database in order to screen out potential biomarkers of gastrointestinal cancers, including GSE20347, GSE92396, GSE103236, and GSE110224, respectively. After analysis using the GEO2R tool and Venn diagram, a total of 21 DEGs were identified: 19 genes were upregulated and 2 genes were downregulated. The Venn diagram is shown in Figure 1A and Figure 1B.

### GO and KEGG Analysis for the DEGs in Gastrointestinal Cancers

For all DEGs, extracellular regions, cell surfaces, the proteinaceous extracellular matrix, and the collagen trimer were mostly enriched in cellular components (CC). The biological process (BP) mainly included the collagen catabolic process, extracellular matrix organization and disassembly, cell adhesion, cartilage development, and skeletal system development. As for molecular function (MF), the DEGs were related to extracellular matrix structural constituent, serine-type endopeptidase activity, metalloendopeptidase activity, protein binding, and extracellular matrix binding (Figure 1C). Furthermore, the KEGG pathway included focal adhesion, ECM-receptor interaction, PI3K-Akt signaling pathway, protein digestion and absorption, and transcriptional mis-regulation in cancer (Figure 1D).

### PPI Network Construction and Modules Analysis

The PPI network of the DEGs was constructed through the STRING online website (Figure 1E). Thus the data were imported into Cytoscape software for visualization and the MCODE plug-in was used to further screen the hub gene. Finally, a total of six hub genes were identified, namely *SPP1, BGN, THBS2, MMP3, COL1A1*, and *TIMP1* (Figure 1F). All the hub genes were upregulated in tumor tissues.

The above results showed that the hub genes were mainly enriched in the extracellular matrix by GO and KEGG, and the protein of *COL1A1* is an important component of the extracellular matrix. Moreover, we found that *COL1A1* was highly expressed in other cancers in addition to gastrointestinal cancers (Grigoroiu et al., 2015; Boguslawska et al., 2016; Liu et al., 2018b; Huang et al., 2019). Therefore, we aimed to explore *COL1A1* in pan-cancer.

### Gene Expression Analysis of *COL1A1* in Pan-Cancer

**FIGURE 2:**
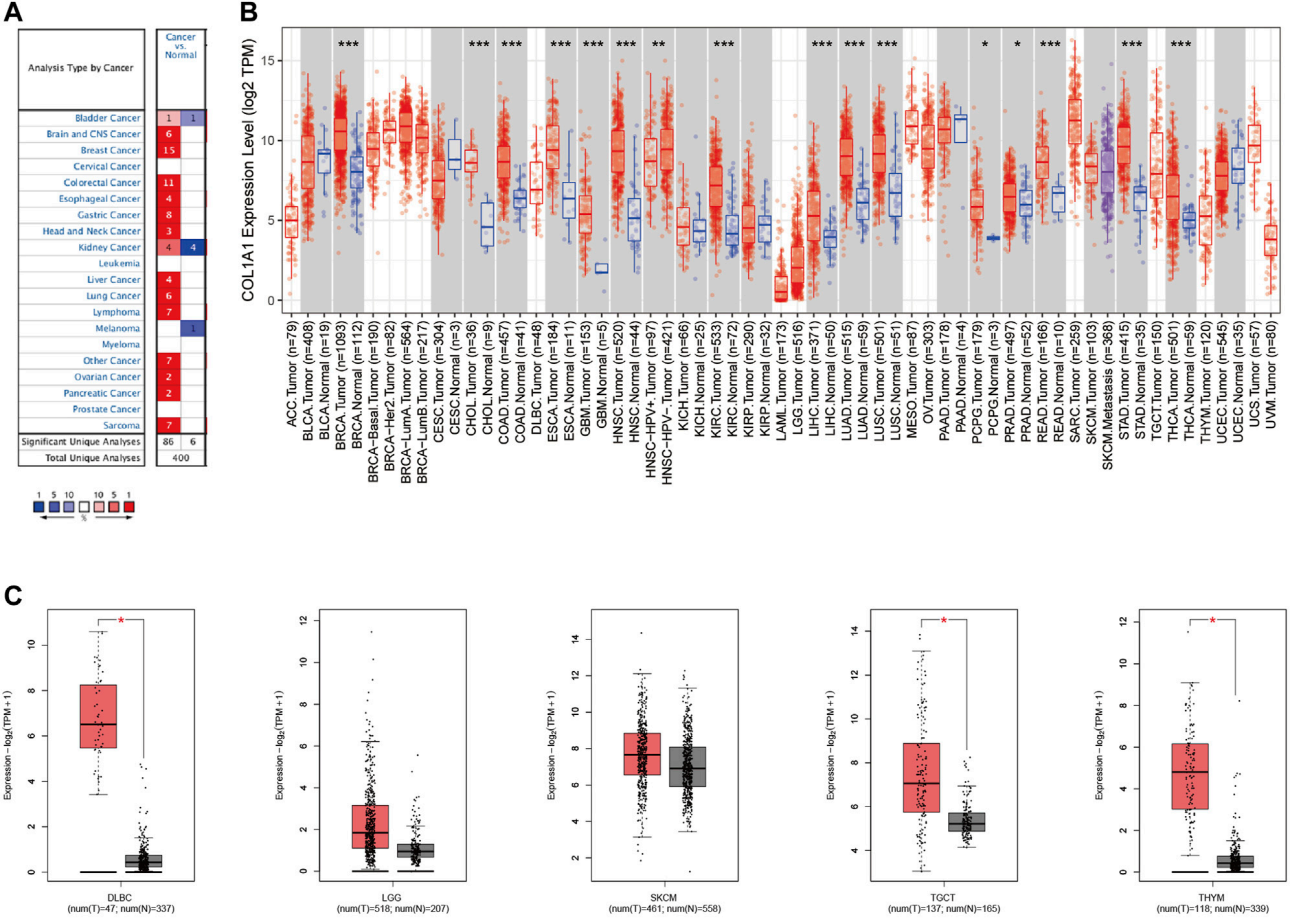
**(A)***COL1A1* expression levels in diverse cancer types and normal tissues by Oncomine. The red color represents increased expression and the blue color represents decreased expression of *COL1A1* in different cancers compared with normal tissues. **(B)** *COL1A1* expression in diverse cancers relative to the non-carcinoma tissue samples based on the TCGA database through TIMER2. *p < 0.05; **p < 0.01; ***p < 0.001. **(C)** *COL1A1* expression in DLBC, LGG, SKCM, TGCT, and THYM by GEPIA2. *p < 0.05.

Through the Oncomine database, we found that *COL1A1* was highly expressed in a variety of tumors (Figure 2A). Moreover, through the TIMER2 database and GEPIA2 database, we found that *COL1A1* was overexpressed mainly in breast invasive carcinoma (BRCA), cholangiocarcinoma (CHOL), colon adenocarcinoma (COAD), esophageal adenocarcinoma (ESCA), glioblastoma multiforme (GBM), head and neck squamous cell carcinoma (HNSC), renal clear cell carcinoma (KIRC), hepatocellular carcinoma (LIHC), lung adenocarcinoma (LUAD), lung squamous cell carcinoma (LUSC), pheochromocytoma and paraganglioma (PCPG), prostate adenocarcinoma (PRAD), rectum adenocarcinoma (READ), stomach adenocarcinoma (STAD), thyroid adenocarcinoma (THCA), diffuse large B lymphoma (DLBC), testicular germ cell tumors (TGCT), and thymoma (THYM) (Figures 2B,C).

We further analyzed the expression of *COL1A1* in different tumor stages. The results showed that *COL1A1* expression is closely related to the late stage of ACC, BCLA, ESCA, KICH, KIRP, STAD, and THCA (Supplementary Figure 1).

### Genetic Alteration Analysis of *COL1A1*

**FIGURE 3:**
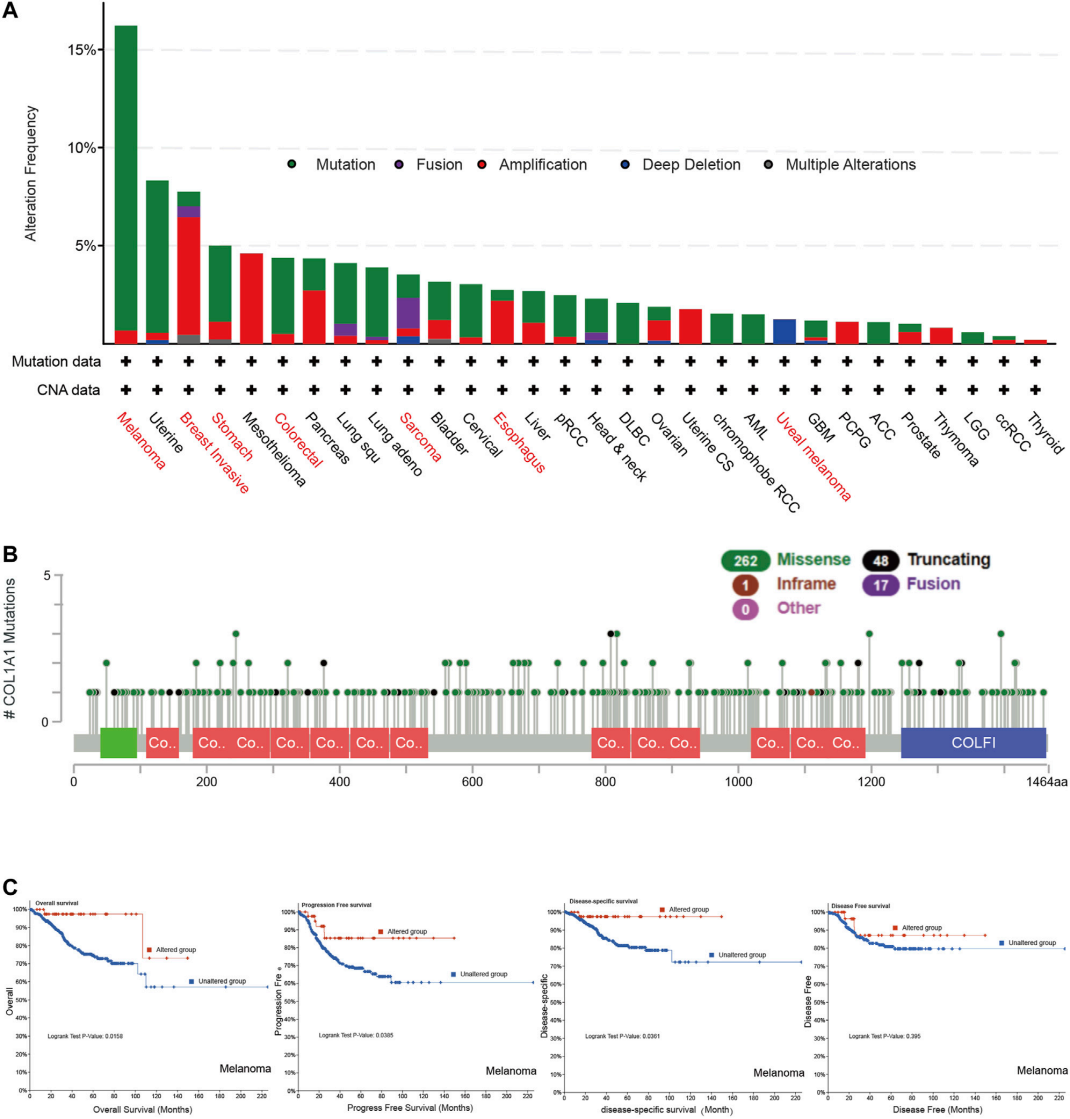
**(A)** Genetic alteration type and frequency of *COL1A1* in different tumors of TCGA by cBioportal. **(B)** Mutation sites of *COL1A1*, which contain 262 missense, 48 truncating, 17 fusion, and 1 inframe mutations. **(C)** *COL1A1* alteration status and overall, progression-free, disease-specific, and disease-free survival of melanoma.

We found *COL1A1* mutation in various tumors by cBioportal. Among them, the highest mutation frequency was in melanoma (16.22%). Breast invasive carcinoma had the highest amplification frequency (6%) and sarcoma had the highest fusion frequency of *COL1A1*. It is noteworthy that all mesothelioma cases had copy number amplification (4.6%) and all uveal melanoma had deletion of *COL1A1* (1.25%) (Figure 3A). The main alteration types of esophageal, gastric, and colorectal cancer were mutation and amplification. Furthermore, Figure 3B presented the types, sites, and case number of the *COL1A1* genetic alteration. The most common alteration type was missense mutation (Figure 3B). In addition, we analyzed the relationship between genetic changes and the prognosis of melanoma. The results showed that the alternations of *COL1A1* are related to the better prognosis of melanoma with overall survival (*p* = 0.0158), progression-free survival (*p* = 0.0385), and disease-specific survival (*p* = 0.0361). Although these alternations showed a tendency of better disease-free survival, it was not statistically significant (*p* = 0.395) (Figure 3C).

### Methylation Profile of *COL1A1*

**FIGURE 4:**
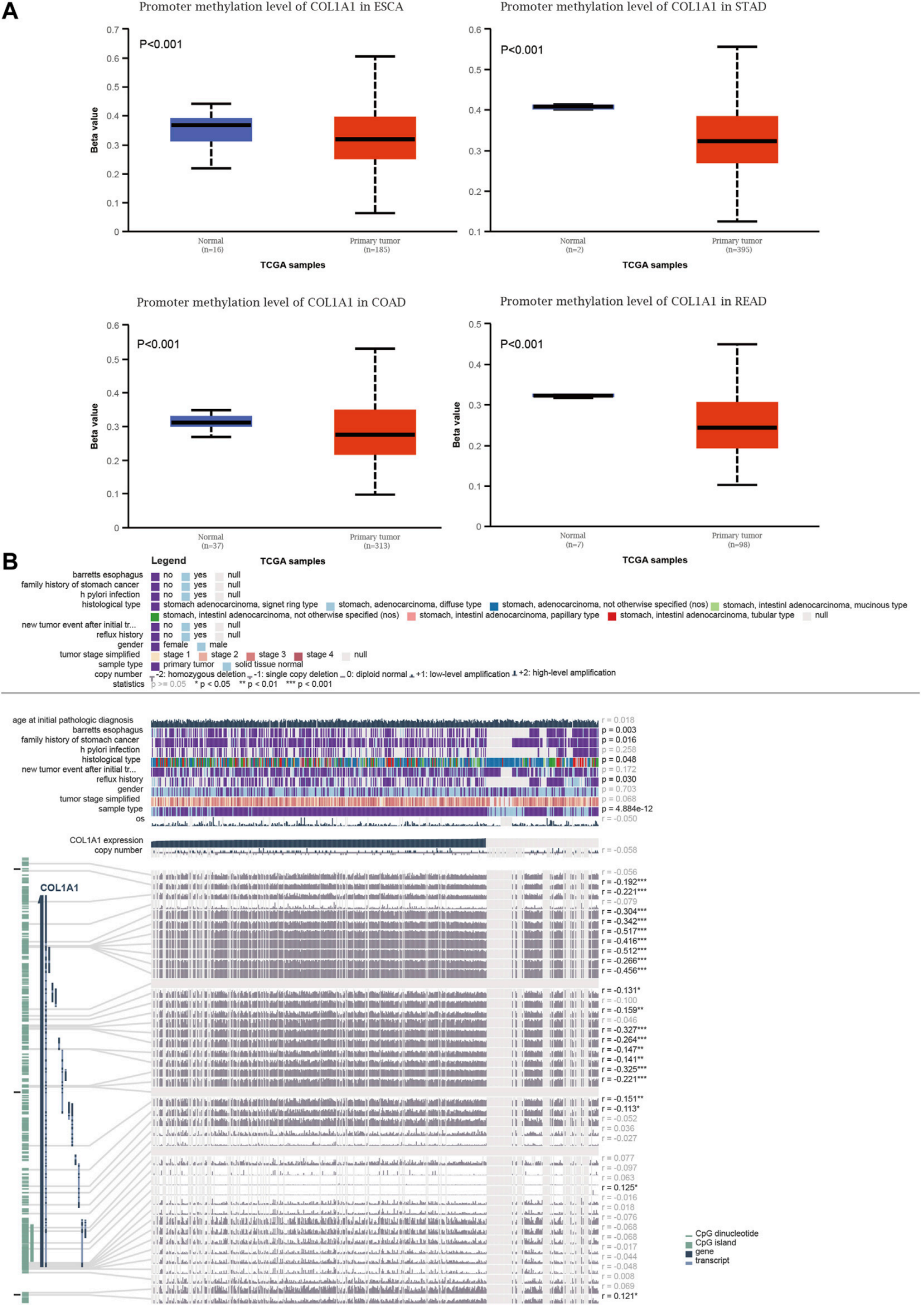
**(A)** Box plots of the *COL1A1* methylation level in normal and gastrointestinal cancers tissues by UALCAN. **(B)** The detailed information of correlation between *COL1A1* expression and DNA methylation, copy number, and clinical data in STAD by MEXPRESS. Copy number of *COL1A1* was examined using Wilcoxon’s rank-sum test and Pearson’s correlation analysis. *p < 0.05, **p < 0.01, ***p < 0.001.

Promoter methylation levels were significantly lower in primary tumors than in normal tissues in gastrointestinal cancers (Figure 4A). The promoter methylation levels of other tumors are shown in Supplementary Figure 2. Moreover, we investigated the detailed information of the correlation between the *COL1A1* expression level and DNA methylation, copy number, and clinical data in STAD (Figure 4B). The results showed that *COL1A1* expression was negatively correlated with DNA methylation of CpG islands (*p* < 0.05, *r* < 0). Furthermore, *COL1A1* expression was closely associated with Barrett’s esophagus (*p* = 0.003), family history (*p* = 0.016), histology type (*p* = 0.048), and reflux history (*p* = 0.030). Copy number variation (CNV) showed no significance in STAD (*r* = −0.058, *p* > 0.05).

### The Relationship Between Survival and Gene Expression of *COL1A1* in Pan-Cancer

**FIGURE 5:**
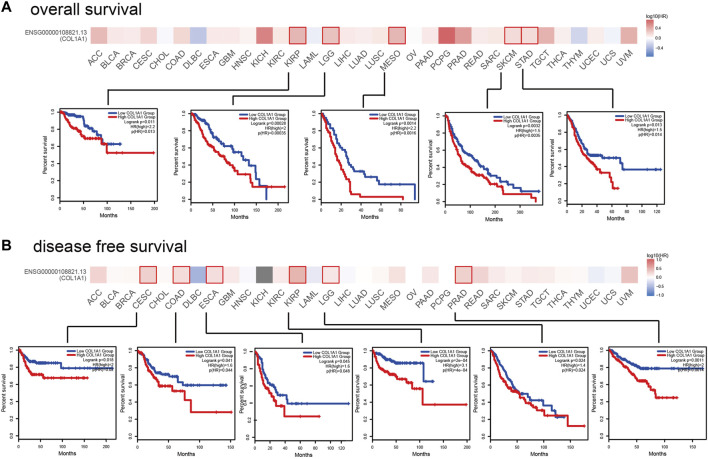
The prognostic impact of *COL1A1* in 33 types of human tumors were examined using GEPIA2. **(A)** High expression of *COL1A1* was associated with poor overall survival in KIRP (HR = 2.2, *p* = 0.011), LGG (HR = 2, *p* = 0.00028), MESO (HR = 2.2, *p* = 0.0014), SKCM (HR = 1.5, *p* = 0.0032), and STAD (HR = 1.5, *p* = 0.013). **(B)** High expression of *COL1A1* was associated with poor disease-free survival in CESC (HR = 2, *p* = 0.018), COAD (HR = 1.6, *p* = 0.041), ESCA (HR = 1.6, *p* = 0.045), KIRP (HR = 3.1, *p* < 0.001), LGG (HR = 1.4, *p* = 0.024), and PRAD (HR = 2, *p* = 0.0011).

In order to analyze the correlation between *COL1A1* gene expression and prognosis, we used GEPIA2 to analyze the OS and DFS of *COL1A1* among different cancers. The results indicated that high expression of *COL1A1* is related to poor overall survival of KIRP (HR = 2.2, *p* = 0.011), LGG (HR = 2, *p* = 0.00028), MESO (HR = 2.2, *p* = 0.0014), SKCM (HR = 1.5, *p* = 0.0032), and STAD (HR = 1.5, *p* = 0.013) (Figure 5A). Moreover, high expression of *COL1A1* is related to poor disease-free survival of CESC (HR = 2, *p* = 0.018), COAD (HR = 1.6, *p* = 0.041), ESCA (HR = 1.6, *p* = 0.045), KIRP (HR = 3.1, *p* < 0.001), LGG (HR = 1.4, *p* = 0.024), and PRAD (HR = 2, *p* = 0.0011) (Figure 5B).

### Immune Infiltration Analysis of *COL1A1* in Pan-Cancer

**FIGURE 6:**
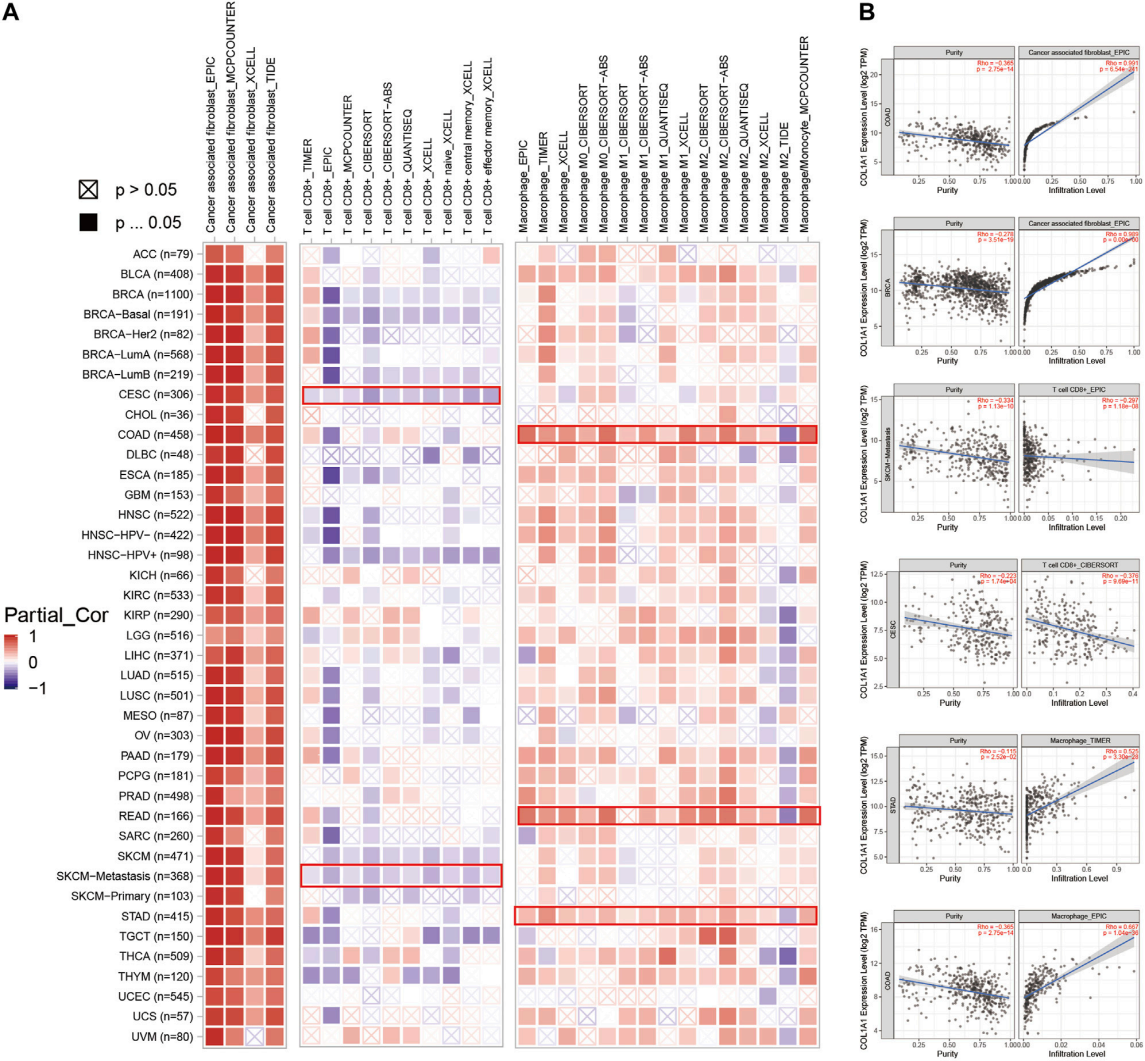
**(A)***COL1A1* expression was positively correlated with cancer-associated fibroblasts in most tumors, and positively correlated with macrophages in BLCA, ESCA, HNSC, COAD, READ, and STAD. However, its expression was negatively correlated with CD8^+^ T cells in CESC, HNSC-HPV+, and SKCM. **(B)** Scatterplot showing the correlation between *COL1A1* expression with purity and infiltration level of cancer-associated fibroblasts (COAD, BRCA), CD 8^+^ T cells (SKCM-metastasis, CESC), and macrophages (STAD, COAD) in certain types of tumors.

In addition, we observed a strong positive correlation between *COL1A1* expression and cancer-associated fibroblasts in most tumors. Moreover, BLCA, ESCA, HNSC, COAD, READ, and STAD showed a statistically positive correlation of *COL1A1* expression and macrophages. However, CESC, HNSC-HPV+, and SKCM showed a negative relationship between *COL1A1* expression and CD8^+^ T cells (Figure 6A). Furthermore, we presented the scatterplot of the correlation between *COL1A1* expression with purity and the infiltration level of cancer associated fibroblast, CD 8^+^ T cells, and macrophages in certain types of tumors (Figure 6B), and the scatterplot showed consistent results with the heatmap.

The results of TISIDB showed that *COL1A1* expression is positively related to TILs in most tumors, especially THCA (Supplementary Figure 3A). Whereas the association of *COL1A1* expression with the MHC molecule (Supplementary Figure 3B), immunoinhibitors (Supplementary Figure 3C), and immunostimulators (Supplementary Figure 3D) is diverse. LGG and THCA showed a positive relation in the MHC molecule. THCA showed a positive relation and TGCT showed a negative relation in most kinds of immunoinhibitors, and TGCT showed a negative relation in most kinds of immunostimulators.

### The Relationship of *COL1A1* Expression and PD-L1 and TMB/MSI in Pan-Cancer

**FIGURE 7:**
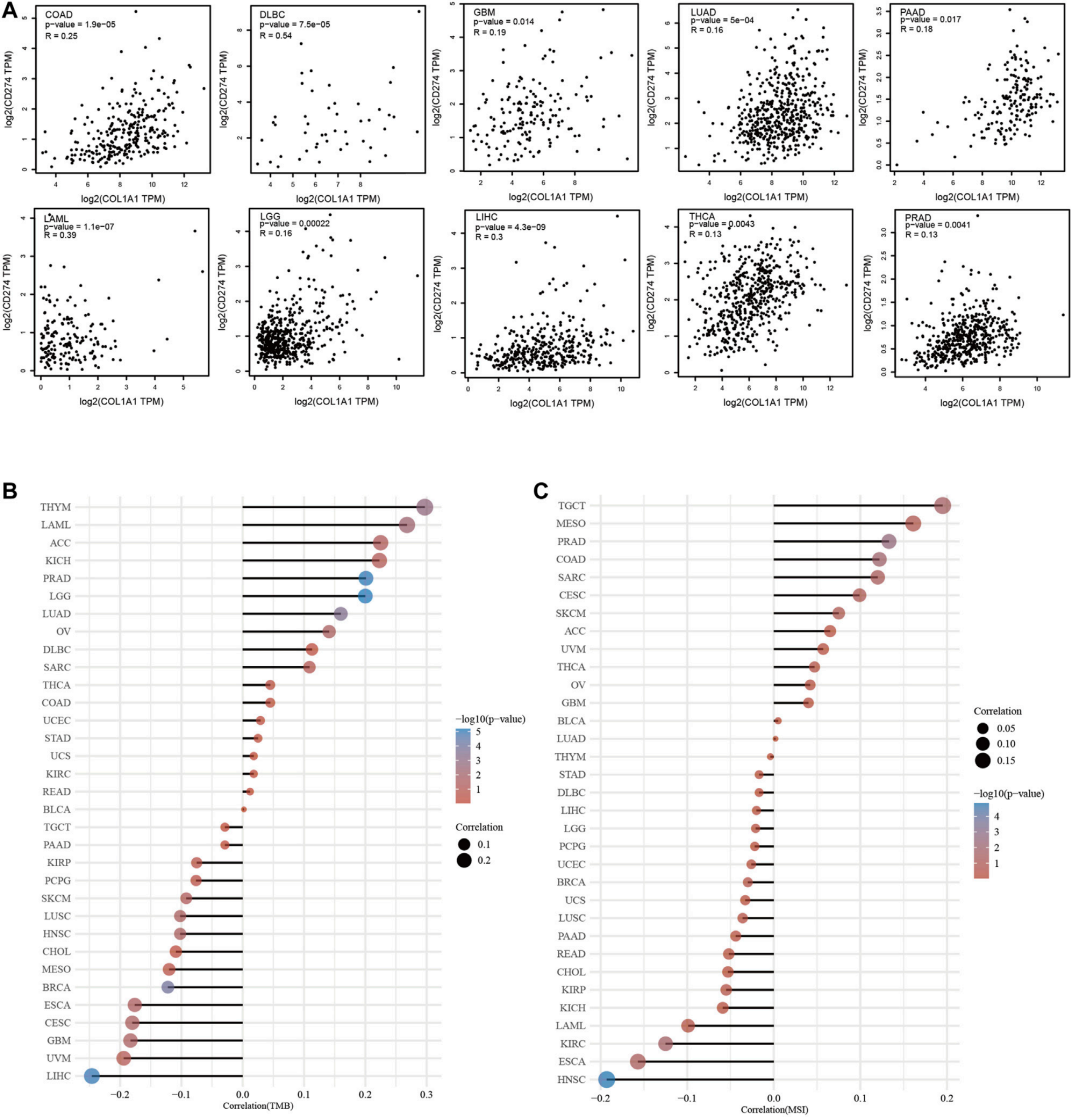
**(A)** The association of *COL1A1* expression and PD-L1 in COAD, DLBC, GBM, LAML, LGG, LIHC, LUAD, PAAD, PRAD, and THCA. **(B)** Spearman correlation analysis of TMB and *COL1A1* gene expression. **(C)** Spearman correlation analysis of MSI and *COL1A1* gene expression. The horizontal axis in the figure represents the correlation coefficient between genes and TMB/MSI, the ordinate is different tumors, the size of the dots in the figure represents the size of the correlation coefficient, and the different colors represent the significance of the *p* value. The bluer the color, the smaller the *p* value.

We found that *COL1A1* expression was positively correlated to PD-L1 in COAD, DLBC, GBM, LAML, LGG, LIHC, LUAD, PAAD, PRAD, and THCA (Figure 7A). Moreover, through analysis of *COL1A1* expression and TMB/MSI, we found that *COL1A1* is closely related to TMB and MSI in a variety of tumors and can be used as a predictor of immunotherapy. Among them, *COL1A1* expression was associated with high TMB in THYM, LAML, ACC, KICH, PRAD, and LGG (Figure 7B). What is more, high correlation of *COL1A1* and MSI was observed in TGCT, MESO, PRAD, COAD, SARC, and CESC (Figure 7C).

### Genes Co-Expressed With *COL1A1* in Pan-Cancer: Pathway and Drug Sensitivity Analysis

**FIGURE 8:**
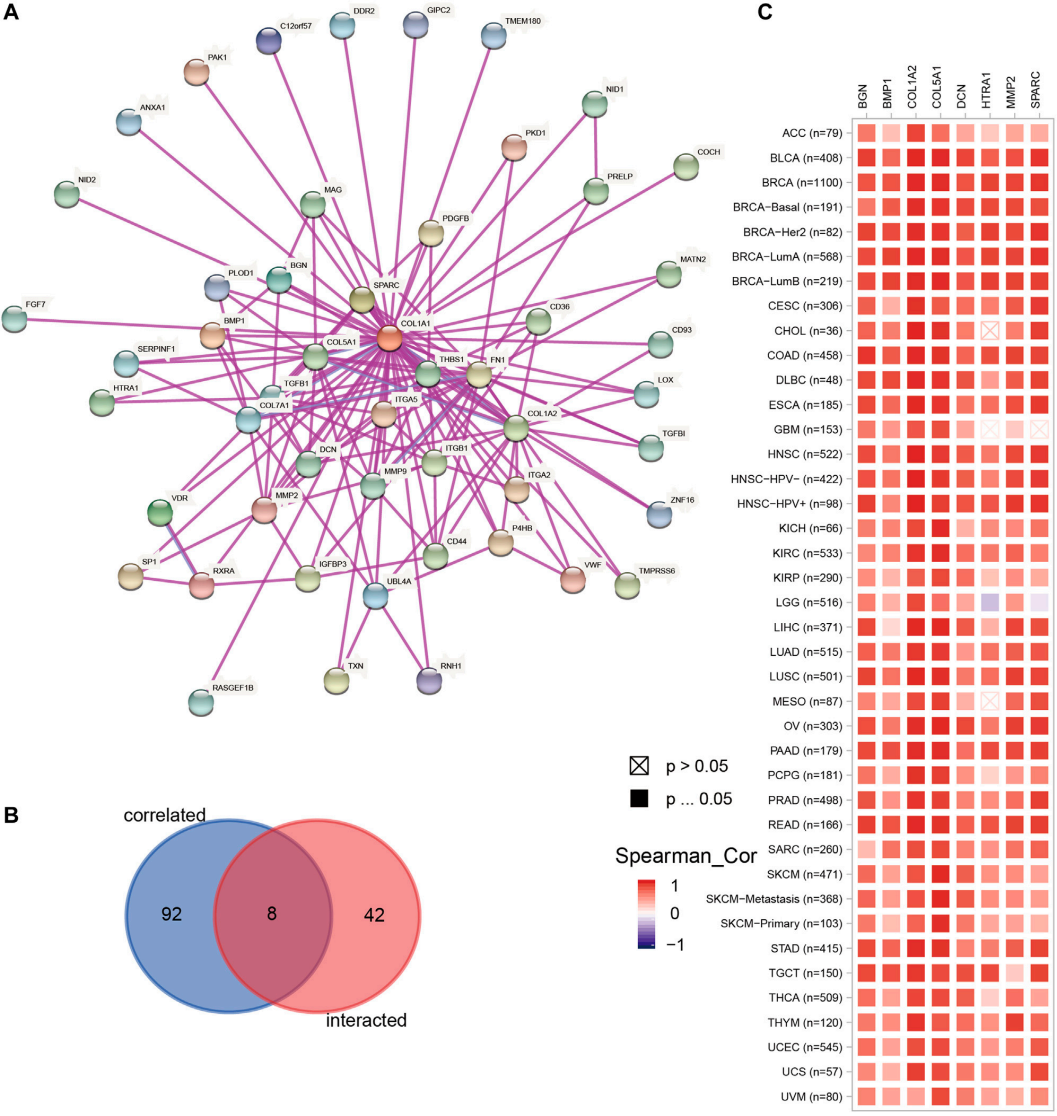
**(A)** The top 50 experimentally determined *COL1A1*-binding proteins using STRING. **(B)** The Venn diagram showing the overlap of the top 50 *COL1A1*-binding proteins and top 100 *COL1A1*-correlated genes in GEPIA2. The overlapping genes were *MMP2*, *SPARC*, *COL5A1*, *DCN*, *BMP1*, *BGN*, *COL1A2*, and *HTRA1*. **(C)** The heatmap confirmed the association of *COL1A1* and *MMP2*, *SPARC*, *COL5A1*, *DCN*, *BMP1*, *BGN*, *COL1A2*, and *HTRA1* in pan-cancer.

**FIGURE 9:**
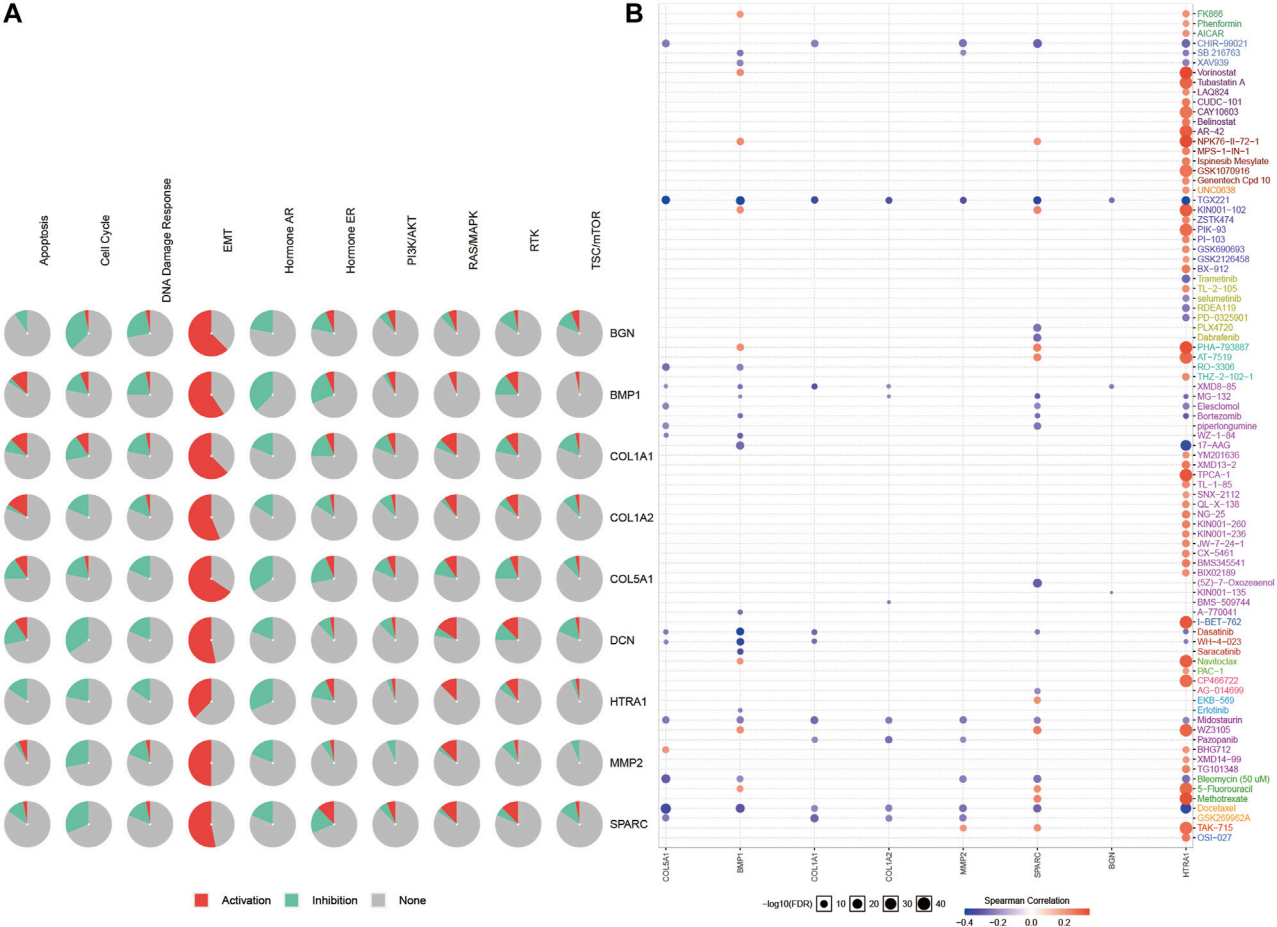
Pathway enrichment **(A)** and drug sensitivity analysis **(B)** of *COL1A1*, *MMP2*, *SPARC*, *COL5A1*, *DCN*, *BMP1*, *BGN*, and *COL1A2* based on GSCALite. The Spearman correlation represented the gene expression that correlated with the drug. Positive correlation means that the high expression of the gene is resistant to the drug, and vice versa.

We obtained 50 experimentally verified proteins related to *COL1A1* based on STRING (Figure 8A). Besides, we obtained 100 genes related to *COL1A1* expression from GEPIA2. Then the Venn diagram showed the overlapping genes (Figure 8B), of which eight genes were closely related to the expression of *COL1A1*, namely, *MMP2*, *SPARC*, *COL5A1*, *DCN*, *BMP1*, *BGN*, *COL1A2*, and *HTRA1*. Heatmaps (Figure 8C) confirmed that these gene expressions were closely related to *COL1A1* among tumors. Then, we performed pathway enrichment and drug sensitivity analysis of *COL1A1* and these eight genes in 33 tumors by GSCALite. The results showed that the pathway was mainly activated in epithelial-mesenchymal transition (EMT) (Figure 9A), and high expression of *HTRA1* was resistant to multiple drugs (Figure 9B).

## Discussion

Recently, several trials reported on the multiple primary tumors of the digestive tract. However, it is difficult to distinguish them from metastatic cancer, thus the diagnosis is relatively complicated. In this study, we screened out four chips of esophageal, gastric, and colorectal cancer from the GEO database and identified 21 DEGs by using the bioinformatics analysis. Furthermore, the PPI network analysis identified six hub genes. All of these genes were upregulated in gastrointestinal cancers. We utilized the methodology of bioinformatics in our previously published studies and successfully screened out some hub genes of certain types of cancer. In this study, we continue to use these same methods and referred to other methods based on some studies.

Moreover, we analyzed one of these six genes, *COL1A1*. It has been reported that the *COL1A1* is highly expressed not only in gastrointestinal cancers but also in other cancers, involving tumorigenesis, metastasis, and immune infiltration. However, there is no pan-cancer research to comprehensively analyze *COL1A1*. Therefore, we expanded the research scope to pan-cancer and analyzed its gene expression level, mutation, DNA methylation, tumor prognosis, tumor immune microenvironment, and the association with predictive markers of immunotherapy, the pathway, and drug sensitivity in 33 kinds of tumors.

*COL1A1* is the main member of the type I collagen family, and the protein is a component of the extracellular matrix. We found that *COL1A1* was highly expressed in various cancers (Ma et al., 2019; Dong et al., 2020; Zhang et al., 2021). The expression of *COL1A1* was negatively correlated with the level of the methylation of its promoter. Hypomethylation of the *COL1A1*’s promoter region might increase its expression level, thereby promoting tumorigenesis. On the other hand, EMT is a dynamic process where inactive polarized epithelial cells transit into active mesenchymal cells (Nieto et al., 2016). *COL1A1* DNA methylation is closely related to the occurrence of EMT (Casalino and Verde, 2020). Researchers have found that the knockdown of *COL1A1* can inhibit the EMT process through the TGF-β signaling pathway, thereby inhibiting the invasion and metastasis of liver cancer and bladder cancer (Ma et al., 2019; Zhu et al., 2019). Thus the methylation of the *COL1A1*’s promoter region might be closely related to the occurrence of EMT. Therefore, *COL1A1* can be viewed as not only the prognostic marker for multiple primary tumors of the digestive tract but also the prognostic marker for pan-cancer.

At present, the efficacy of immunotherapy is still not satisfied in many cancers, and patients might experience immunoresistance during the treatment. The results of immune checkpoint inhibitors (ICIs) are closely related to the tumor immune microenvironment and the expression levels of PD-L1, TMB, and MSI. An impressive finding of this study was that the expression of *COL1A1* was positively correlated with the abundance of CAFs in almost all kinds of cancers, and macrophage infiltration in gastrointestinal cancers, BLCA, and HNSC, whereas it was negatively correlated with the quantity of CD8^+^ T cells in CESC, HNSC-HPV+, and SKCM, respectively. Moreover, its expression is also related to the levels of PD-L1, TMB, and MSI. It is reported that the relationship between immune cells and PD-L1 is complicated. On the one hand, CAFs and macrophages induced CD8^+^ T cell deletion (Lakins et al., 2018; Dong et al., 2021). On the other hand, CAFs promote the expression of PD-L1 in tumor cells by secreting CXCL2 and CXCL5 (Inoue et al., 2019; Li et al., 2019), and macrophages upregulate PD-L1 expression through TGF-β–induced EMT (Vakili-Ghartavol et al., 2018; Jiang and Zhan, 2020), which plays a critical role in tumor immunosuppression and immune evasion. This might be helpful to understand the mechanisms of immunoresistance. Furthermore, Peng et al. (2020) found that collagen promotes ICIs resistance through LAIR1-dependent CD8^+^ T cell exhaustion. Thus, it is worth studying whether *COL1A1*, a member of the collagen family, causes resistance of ICIs through LAIR1-dependent CD8^+^ T cell exhaustion or other mechanisms. In addition, we hypothesized that if the expression of *COL1A1* or relative pathways were inhibited, CD8^+^ T cells might be enriched in the tumor microenvironment, and the number of CAFs, macrophages, and the PD-L1 expression level might decrease, resulting in attenuated immunoresistance.

The FDA pooled analysis showed that the efficacy of immunotherapy combined with chemotherapy was better than that of immunotherapy alone (mPFS 7.7 vs. 4.2 months) for the first-line treatment of advanced non-small cell lung cancer with 1–49% of the PD-L1 expression level (Akinboro et al., 2021). If high expression of *COL1A1* was detected in these patients, we hypothesize that adding the *COL1A1* inhibitor to ICIs plus chemotherapy would further raise the efficacy, and relevant clinical trials could be carried out to study its efficacy and toxicity in the future. Another phase II study compared the sequence of chemotherapy and anti–PD-1 antibody in neoadjuvant therapy for locally advanced esophageal squamous cell carcinoma. The results showed that immunotherapy given 2 days after chemotherapy had a higher pathological complete response rate (36.4 vs. 7.7%) compared with simultaneous administration (Zhao et al., 2021). This result might be explained by the killing of activated T cells caused by chemotherapy. For cancers with high *COL1A1* expression, we found a low infiltration of CD 8^+^ T cells by TIMER2. Future experiments could explore whether the efficacy might be better if the *COL1A1* inhibitor was given before ICIs.

The combination of radiotherapy and immunotherapy might be another research highlight. It is believed that radiation could activate the immune system and reverse cold tumors into hot tumors, and thus improve the efficacy of immunotherapy. However, the results of the FORCE study showed that nivolumab combined with radiotherapy (5 × 4 Gy) did not increase the objective response rate of advanced non-small cell lung cancer compared to nivolumab alone (Bozorgmehr et al., 2020). For these patients, whether the efficacy of combination therapy can be enhanced if the *COL1A1* inhibitor were administrated might be worthy of further discussion.

It was found that the efficacy of EGFR-TKI was not satisfied for NSCLC patients with a high level of PD-L1, and drug resistance may appear during the treatment (Su et al., 2018). Yamazaki et al. (2018) found that type I collagen induces EGFR-TKI resistance by activating mTOR. Under this condition, we can further study whether EGFR-TKI combined with an mTOR inhibitor or *COL1A1* inhibitor can overcome EGFR-TKI resistance.

In the era of precision medicine, it would be exciting to carry out a biomarker-guided basket trial to study the potential role of *COL1A1* inhibitors in different cancers, and it might be an optional treatment for patients with advanced tumors, just like pembrolizumab could be used in MSI-H patients.

Nevertheless, although we employed multiple bioinformatics databases to analyze the role of *COL1A1* across 33 tumors, this study still has some limitations. Firstly, the current study merely focused on *COL1A1* at the transcriptional level, but lacked the description of its protein transcriptome and the post-translational level. Secondly, the results of our study lack external validation in other public datasets. Finally, the databases used in this study were mainly based on the gene-chip and sequencing data of tumor tissue from TCGA, so the cell-level analysis of immune cell markers could have introduced systematic bias. Future studies should focus more on single-cell sequencing technology, *in vitro* experiments, and translational research.

## Conclusion

**FIGURE 10:**
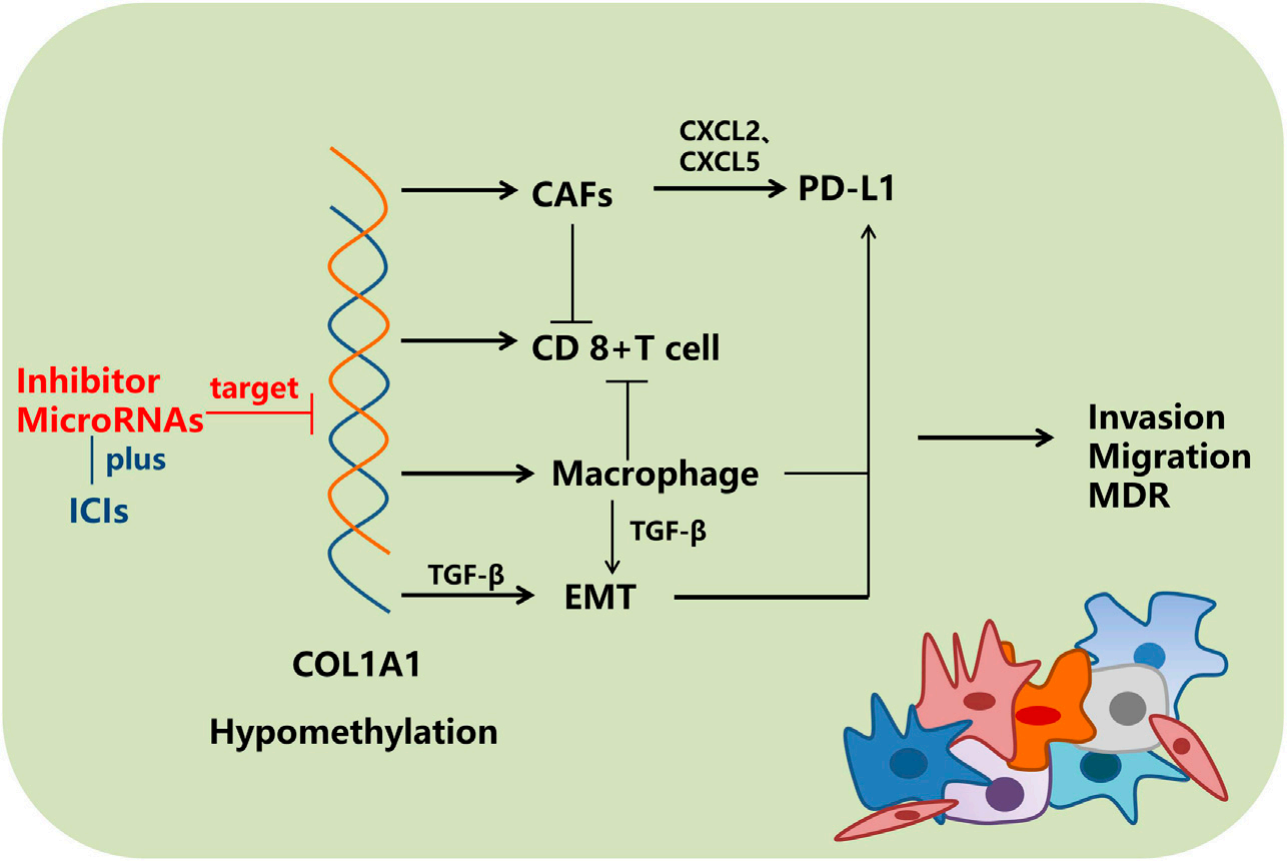
Overview of the regulatory mechanism of *COL1A1.* MDR: multidrug resistance.

In conclusion, we found that *COL1A1* could be a marker for different cancers and is critical for tumor immune microenvironments (Figure 10). Further exploration of the function of *COL1A1* might provide additional information on precision oncology.

## Data Availability

The datasets presented in this study can be found in online repositories. The names of the repository/repositories and accession number(s) can be found in the article/Supplementary Material.
