# Supplementary figures and images for: Prognostic Prediction, Immune Microenvironment, and Drug Resistance Value of Collagen Type I Alpha 1 Chain: From Gastrointestinal Cancers to Pan-Cancer Analysis
Source: Front Mol Biosci. 2021 Jul 30;8:692120. doi: 10.3389/fmolb.2021.692120 (PMC8361495; doi:10.3389/fmolb.2021.692120)

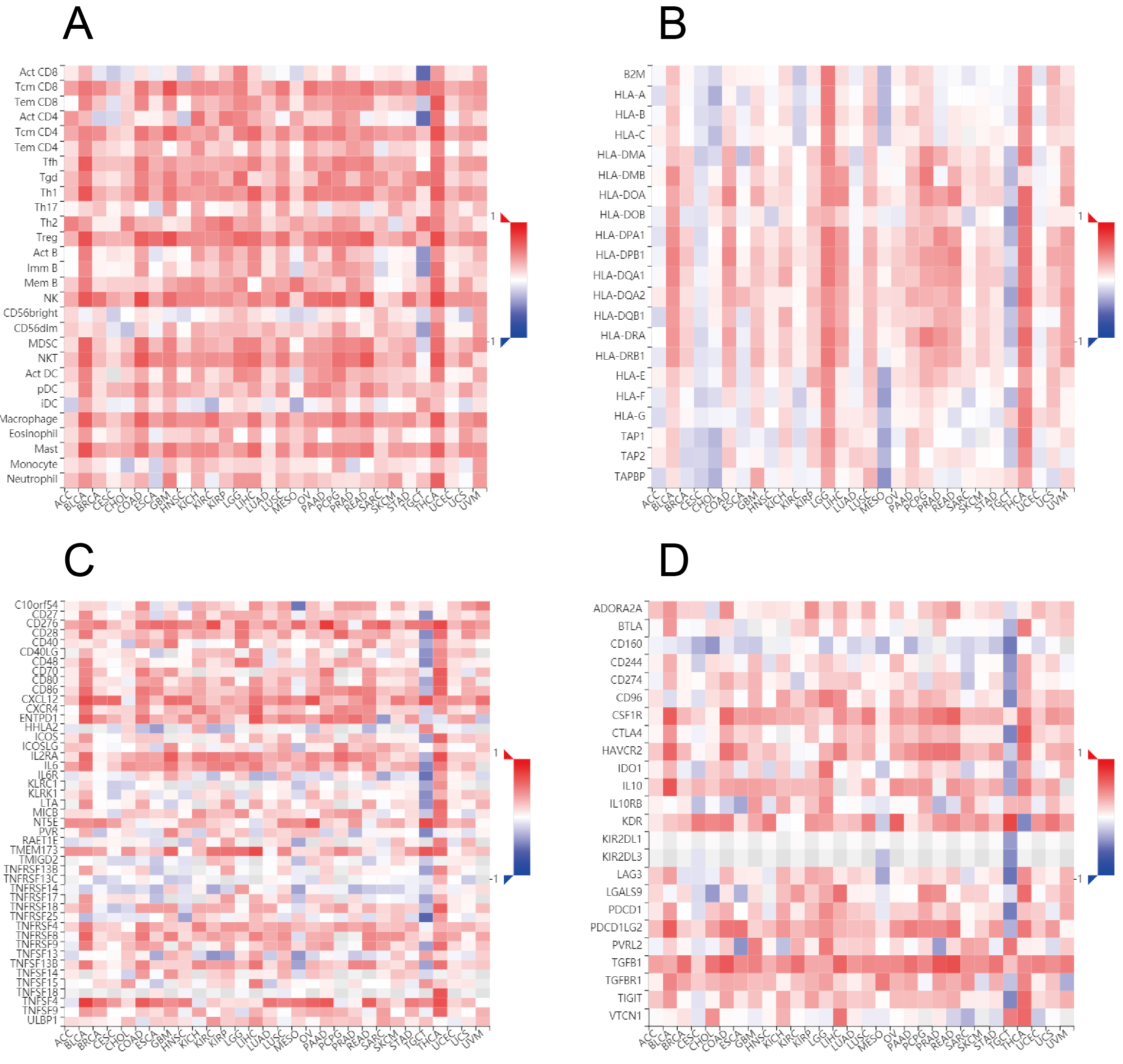

Supplement: Supplementary file 1 [file Image3.jpg]

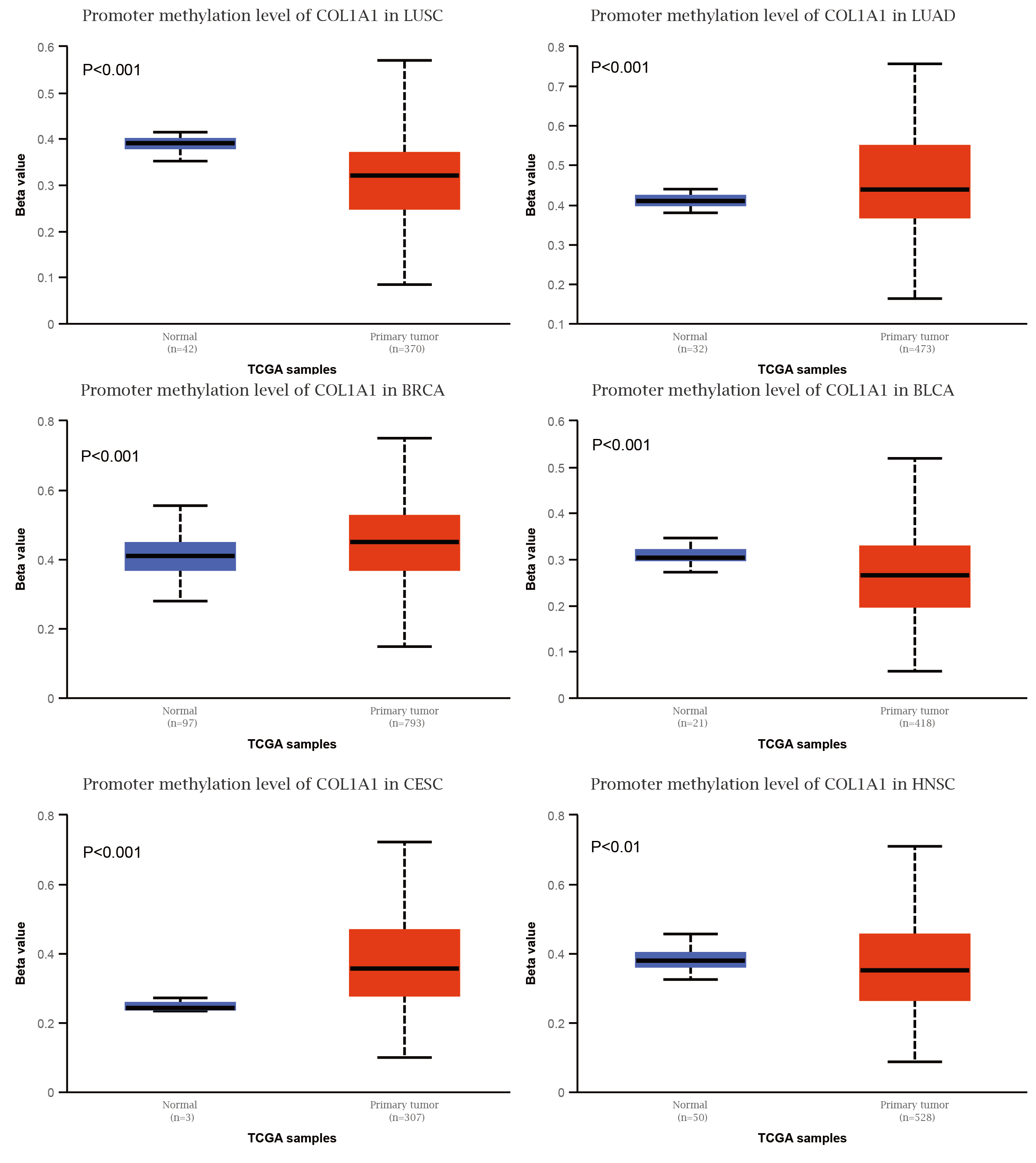

Supplement: Supplementary file 2 [file Image2.jpg]

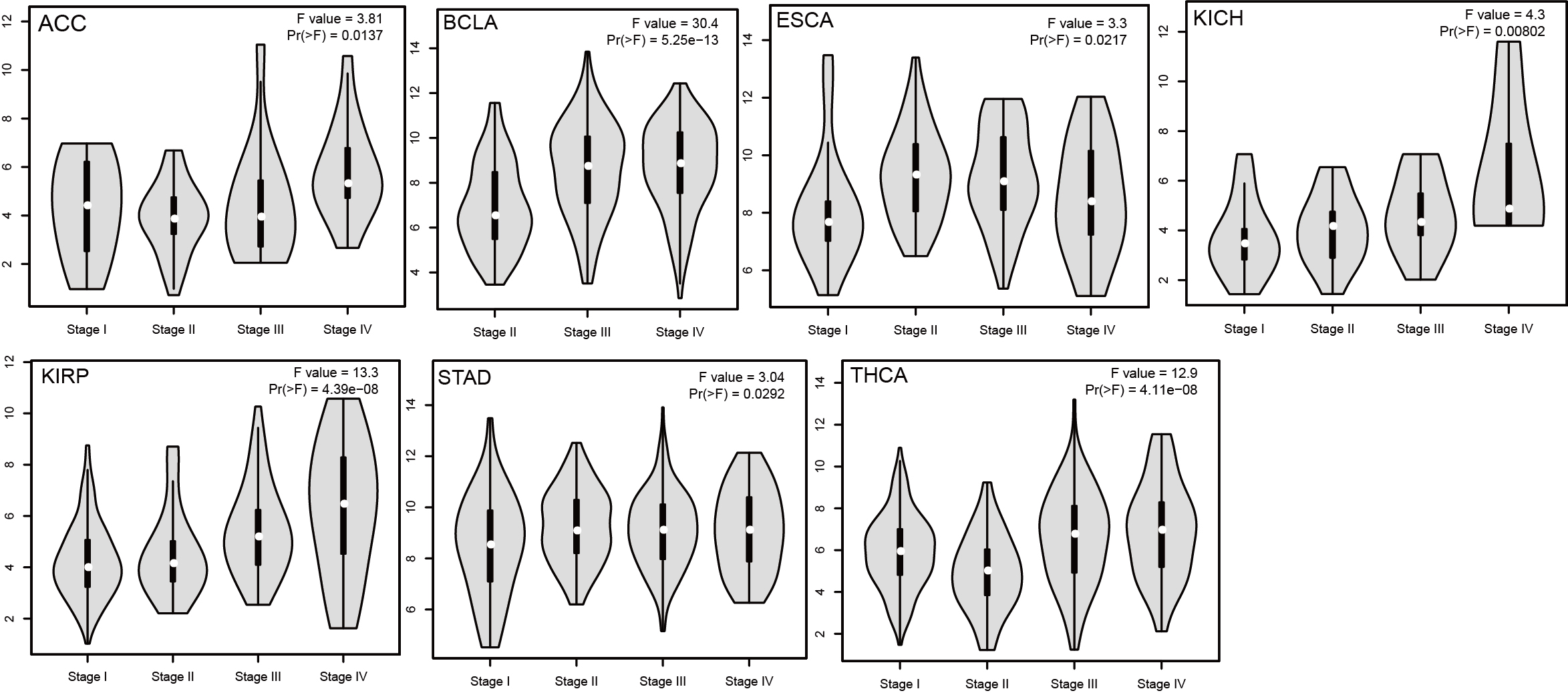

Supplement: Supplementary file 3 [file Image1.JPEG]
